# Auditory and academic skills self-perception in adults

**DOI:** 10.1590/2317-1782/20242023098en

**Published:** 2024-06-14

**Authors:** Bruna Stéfanie Pereira, Luciana Macedo de Resende, Luciana Cássia de Jesus, Andrezza Gonzalez Escarce, Luciana Mendonça Alves

**Affiliations:** 1 Departamento de Fonoaudiologia, Faculdade de Medicina, Universidade Federal de Minas Gerais – UFMG - Belo Horizonte (MG), Brasil.

**Keywords:** Auditory Perception, Learning, Hearing, Speech, Language and Hearing Sciences, Students, Self Report, Adult

## Abstract

**Objective:**

To describe and analyze auditory and academic complaints of students and employees of a federal public university.

**Methods:**

The study was carried out using a non-probabilistic. The EAPAC Scale with adaptations was used to fulfill the research objectives. It has 14 questions about complaints related to listening skills and 12 questions related to the academic environment. Descriptive data analysis was performed through the frequency distribution of categorical variables and Pearson's chi-square test was used for association analyses.

**Results:**

646 individuals aged between 17 and 67 years old participated in the research. The most prevalent complaints were academic difficulty related to memory, concentration, and planning, hearing and understanding speech in noise, and memorization of tasks that were only heard. There was an association with bidirectional statistical significance between academic and auditory complaints.

**Conclusion:**

It was possible to observe that there is an association between auditory and academic complaints in adults, marked by the relationship between cognitive and auditory aspects. It is relevant that these factors are considered when performing assessments of Central Auditory Processing when intervening in patients with auditory complaints, and in student life.

## INTRODUCTION

Hearing puts individuals in contact with their environment and is highly relevant to the development of learning, as it allows them to analyze and understand auditory information in different contexts^(1)^.

Central auditory processing (CAP) encompasses a set of skills, such as identifying the lateralization and spatial location of the sound; understanding speech in noise; understanding a distorted and fragmented message; directing attention to stimuli presented to one ear, to the detriment of sounds presented to the opposite ear; recognizing different sounds presented to both ears simultaneously; discriminating small changes in sound stimuli; and detecting and perceiving modulations and minimum intervals in a sound sequence^(2)^.

Thus, central auditory skills enable the person to recognize spoken words, music, noises, and environmental sounds; distinguish voiceless from voiced phonemes of the mother tongue; recognize similar phonemes; and integrate and order sounds to record auditory information^(3)^. They also assist in prosody and phonological and syntactic organization^(3)^.

In addition to these skills, auditory attention, memory, and comprehension are essential for processing sound stimuli. Auditory attention plays an important role in language reception and expression^(3)^, while auditory memory helps retain auditory information^(4)^, and listening comprehension allows one to understand the meaning of auditory information associated with the other auditory skills mentioned^(5)^.

Thus, there is an evident relationship between auditory skills and the learning process, since to learn it is necessary to discriminate, recognize, and retain sounds, establish their meanings and definitions, and integrate auditory information with the other senses to understand melodic aspects of speech and information with double meaning or whose meaning needs to be inferred^(6,7)^.

CAP can be assessed either behaviorally – through a battery of important tests to analyze the functioning of auditory skills^(2)^ and guide therapeutic processes – or electrophysiologically^(2)^. The literature has also mentioned using questionnaires complementarily to identify signs and symptoms of risk for central auditory processing disorder (CAPD)^(8)^. These instruments are low-cost, simple to fill out and collect relevant information about auditory behavior in a naturalistic environment, as professionals cannot verify such data in environments other than the office^(8-10)^.

Due to the scarcity of CAP skills screening instruments for Brazilian adults^(11)^, the Central Auditory Processing Skill Self-Perception Scale (CAPSSPS)^(11)^ was developed for young adults. It aims to identify auditory skills at risk of changes and enable the development of strategies to promote hearing health and learning^(10)^ in this population. The scale consists of questions related to hearing and learning complaints. A study^(11)^ aimed to validate this scale, concluding through psychometric analysis that the CAPSSPS items had a good correlation with their valid results, demonstrating its reliability and enabling inferences about possible changes in auditory processing.

Hence, the following questions arise: What would the most prevalent auditory and academic complaints be in university students and former university students? Would there be an association between auditory and academic complaints in this group? Studies in the literature have addressed the influence of auditory processing on learning difficulties in children, with positive correlations between the two aspects^(12-14)^. However, there are still gaps on the topic with the adult target audience.

Therefore, this study aimed to describe and analyze auditory and academic complaints of university students, obtained through this group's responses to the CAPSSPS^(11)^ with adaptations made by the authors.

## METHOD

This descriptive, comparative, cross-sectional study was approved by the Research Ethics Committee of the Federal University of Minas Gerais (UFMG) under evaluation report no. 5.137.573. All participants were informed about the study objectives and procedures and signed an informed consent form.

Students and employees from a federal public university were invited to participate in the research via email sent by the university's Information Technology Department. The invitation was sent to the institutional email of around 50,561 individuals, with a link to Google Forms with the informed consent form and the CAPSSPS^(11)^ with adaptations made by the authors. They included seven questions in the auditory domain and five in the academic domain, as they may be associated with auditory complaints.

The CAPSSPS^(11)^ with adaptations (Chart 1) has 26 questions – 25 closed-ended ones about the person’s difficulties and one open-ended question. The scale has 14 questions on auditory skills complaints (sound source detection, localization, and lateralization; recognition, discrimination, and selective and sustained attention; short-term auditory memory and temporal aspects of hearing) and 12 questions related to the academic environment (concentration, memory, planning, and learning).

**Chart 1:** Central Auditory Processing Skill Self-Perception Scale (CAPSSPS) with adaptations

CAPSSPS - CENTRAL AUDITORY PROCESSING SKILL SELF-PERCEPTION SCALE IN ADULTS: EXPANDED		ANSWERS	
Name:__________________________________________ Sex: __________________ Age:____________________ Education level:_______________________________________________________ Phone: ( ) ___________ - ____________ Date:_____/_____/_________ **Where did you go to high school? ( ) public school ( ) private school ( ) not applicable**		**Yes**	No
Auditory domain Academic domain		SCORE	
QUESTIONS		**(1)**	(0)
**Q1**	Do you believe you have problems detecting sound (sound in general, speech, or other sounds)?		
**Q2**	Do you believe you have problems with sound source location and lateralization (for example, knowing where someone is calling from at a distance)?		
**Q3**	Do you believe you have problems identifying sounds in general?		
**Q4**	Do you believe you have problems with sound discrimination (differentiating speech sounds – for example, hearing S and Z)?		
**Q5**	Do you believe you have problems with selective and sustained attention to sound (for example, listening to and understanding the professor's speech, even if there is another conversation in the room or external noise)?		
**Q6**	Do you believe you have short-term memory problems related to sound (remembering things you have only heard, such as short texts and lectures)?		
**Q7**	Do you believe you have difficulty perceiving sounds in time? For example, understanding someone who speaks too quickly or articulates words unclearly.		
**Q8**	Do you believe you have difficulty hearing and understanding speech in noisy situations? For example, talking at the bus stop, in restaurants, etc.		
**Q9**	Do you have or have you had academic difficulties related to concentration at any point in your academic life or professional activity?		
**Q10**	Do you have or have you had academic difficulties related to memory at any point in your academic life or professional activity?		
**Q11**	Do you have or have you had academic difficulties related to planning at any point in your academic life or professional activity?		
**Q12**	Do you have or have you had academic difficulties related to learning at any point in your academic life or professional training?		
**Q13**	Do you have difficulty understanding the information you read?		
**Q14**	Do you have difficulty understanding information that the author did not write in the text and that needs to be deduced (which is between the lines)?		
**Q15**	Do you change letters that represent similar sounds when writing or reading? Below are some letters that represent similar sounds: B – P, D – T, G – C/Q/K, V – F, Z – S, J – X/CH. E.g.: Cola – Gola, Já – Chá, Vaca – Faca		
**Q16**	Do you have difficulty making and noticing pauses in the text according to punctuation marks?		
**Q17**	Are you fluent in any foreign language?		
**Q18**	Do you study or have you studied any foreign language? If so, which one and for how long? If you do not study or have not studied, answer no.		
**Q19**	Do you have difficulty learning a new language?		
**Q20**	Do you have difficulty perceiving when someone wants to give a different meaning to the information being said by changing their tone of voice?		
**Q21**	Do you have difficulty understanding jokes or words with double meanings?		
**Q22**	Do you have difficulty perceiving and reproducing rhythms?		
**Q23**	If you are talking to someone and don't hear a part of what they said, do you have difficulty understanding the entire message/speech?		
**Q24**	Do you have difficulty memorizing tasks and arrangements that were just heard (without taking notes)?		
**Q25**	Do you have difficulty finishing complex activities (which require formulating and giving responses) by the deadline?		
**Q26**	Do you have difficulty following tasks with varied stimuli, such as sounds, images, texts, and animation?		

The study also collected data on sociodemographic characteristics (name, age, sex, place of birth, education, level, and type of high school they had attended).

The study had a non-probabilistic sample, initially consisting of 697 individuals from the said university, who responded to the CAPSSPS^(11)^ with adaptations.

The inclusion criterion was to be associated with the university as a student or employee and, as an exclusion criterion, not having at least an incomplete higher education level, as the aim was to characterize the academic and hearing difficulties of individuals who attended university. Therefore, 51 participants were excluded from the study, as they had only attended high school, totaling a sample of 646 members.

The participants were divided into four groups, namely: with auditory and academic complaints (comprising 538 people), with academic complaints (39 people), with auditory complaints (45 people), and without complaints (24 people).

The study performed descriptive analysis of the data, using the frequency distribution of the categorical variables.

Pearson's Chi-square test was used for association analysis, setting statistical significance at p-value ≤ 0.05.

After inserting the data into an Excel^®^ spreadsheet, they were entered, processed, and analyzed in SPSS software, version 25.0.

## RESULTS

Table 1 describes the general characteristics of the study participants. One participant obtained a high school diploma through a specific authorized test – to whom the question on the type of high school they had attended did not apply.

**Table 1:** Descriptive analysis of the general characteristics of the sample

Data	Variables	Values
Age	Minimum	17
	Maximum	67
	Median	29
	Under 30 years n(%)	328(50.8)
	30 years or older n(%)	318(49.2)
Sex	Males n(%)	208(32.2)
	Females n(%)	438(67.8)
Nationality	Brazilian n(%)	645(99.8)
	Foreigner n(%)	1(0.2)
Education level	Higher education incomplete n(%)	292(45.2)
	Higher education degree n(%)	110(17.0)
	Postgraduation n(%)	86(13.3)
	Master’s degree n(%)	120(18.6)
	Doctoral degree n(%)	38(5.9)
High school	Public school n(%)	400(61.9)
	Private school n(%)	245(37.9)
	Not applicable n(%)	1(0.2)
Complaints	No complaints n(%)	24(3.7)
	Auditory and academic complaints n(%)	538(83.3)
	Auditory complaints n(%)	45(7.0)
	Academic complaints n(%)	39(6.0)

**Caption:** n = number of participants

Most research participants were females (67.8%), aged 17 to 67 years, Brazilian (99.8%), with incomplete higher education (45.2%), with both auditory and academic complaints (83.3%), and had attended a public high school (61.9%).

**Figure 1:**
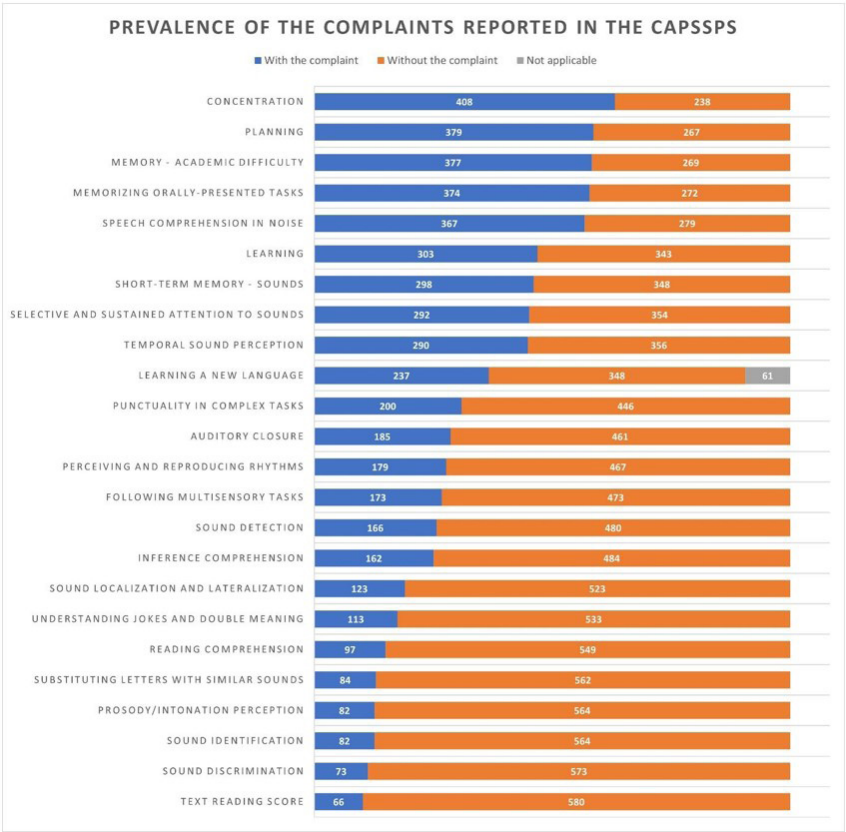
Chart with the prevalence of auditory and extra-auditory complaints reported in the questionnaire

Figure 1 shows a prevalence of auditory and academic complaints reported in the questionnaire. The most prevalent complaints were academic difficulties related to concentration (63.16%), planning (58.67%), memory (58.36%), memorizing tasks that were only heard (57.9%), and hearing and understanding speech in noise (56.8%).

An association analysis was performed between the most reported complaints and the other ones. Complaints of academic difficulties related to concentration, memory, and planning were associated with auditory domain questions, while complaints of auditory difficulties related to hearing and understanding in noisy environments and memorizing tasks and arrangements only heard were associated with academic domain questions. This division aimed to verify whether the participants' auditory and academic complaints were associated.

Table 2 highlights the associations between difficulty hearing and understanding in noisy environments and academic difficulties, using Pearson's chi-square test. This analysis found a statistically significant association between difficulty hearing and understanding in noisy environments and having or having had academic difficulties related to memory (p ≤ 0.001). Also, those who did not have difficulty in noisy environments tended not to have difficulties in learning (p = 0.027), understanding written information (p = 0.008), understanding information that needs to be deduced (p = 0.028), or carrying out complex tasks on time (p = 0.005).

**Table 2:** Association between difficulties in speech comprehension in noise, memorizing tasks that were only heard, and academic difficulties

Variables		Difficulty with noise environments			Difficulty in memorizing tasks that were only heard		
		Yes	No	p-value	Yes	No	**p-value**
		n (%)	n (%)		n (%)	n (%)	
Academic difficulties related to concentration	Yes	241 (65.7)	136 (48.7)	≤0.001*	265 (70.9)	143 (52.6)	≤0.001*
	No	126 (34.3)	153 (51.3)		109 (29.1)	129 (47.4)	
	Total	367 (100.0)	279 (100.0)		374 (100.0)	272 (100.0)	
Academic difficulties related to memory	Yes	241 (65.7)	136 (48.7)	≤0.001*	262 (70.1)	115 (42.3)	≤0.001*
	No	126 (34.3)	153 (51.3)		112 (29.9)	157 (57.7)	
	Total	367 (100.0)	279 (100.0)		374 (100.0)	272 (100.0)	
Academic difficulties related to planning	Yes	227 (61.9)	152 (54.5)	0.059	245 (65.5)	134 (49.3)	≤0.001*
	No	140 (38.1)	127 (45.5)		129 (34.5)	138 (50.7)	
	Total	367 (100.0)	279 (100.0)		374 (100.0)	272 (100.0)	
Academic difficulties related to learning	Yes	186 (50.7)	117 (41.9)	0.027*	205 (54.8)	98 (36.0)	≤0.001*
	No	181 (49.3)	162 (58.1)		169 (45.2)	174 (64.0)	
	Total	367 (100.0)	279 (100.0)		374 (100.0)	272 (100.0)	
Difficulties understanding written information	Yes	67 (18.3)	30 (10.8)	0.008*	72 (19.3)	25 (9.2)	≤0.001*
	No	300 (81.7)	249 (89.2)		302 (80.7)	247 (90.8)	
	Total	367 (100.0)	279 (100.0)		374 (100.0)	272 (100.0)	
Difficulties understanding non-written/deduced information	Yes	104 (28.3)	58 (20.8)	0.028*	120 (32.1)	42 (15.4)	≤0.001*
	No	263 (71.7)	221 (79.2)		254 (67.9	230 (84.6)	
	Total	368 (100.0)	279 (100.0)		374 (100.0)	272 (100.0)	
Substituting letters with similar sounds in writing or reading	Yes	52 (14.2)	32 (11.5)	0.312	54 (14.4)	30 (11.0)	0.203
	No	315 (85.8)	247 (88.5)		320 (85.6)	242 (89.0)	
	Total	367 (100.0)	279 (100.0)		374 (100.0)	272 (100.0)	
Difficulties in making/perceiving pauses in the text according to punctuation	Yes	44 (12.0)	22 (7.9)	0.088	45 (12.0)	21 (7.7)	0.074
	No	323 (88.0)	257 (92.1)		329 (88.0)	251 (92.3)	
	Total	367 (100.0)	279 (100.0)		374 (100.0)	272 (100.0)	
Difficulties learning a new language	Yes	141 (42.3)	96 (38.1)	0.300	151 (43.5)	86 (36.1)	0.075
	No	192 (57.7)	156 (61.9)		196 (56.5)	152 (63.9)	
	Total	333 (100.0)	252 (100.0)		374 (100.0)	272 (100.0)	
Difficulties in finishing complex activities	Yes	130 (35.4)	70 (25.1)	0.005*	151 (40.4)	49 (18.0)	≤0.001*
	No	237 (64.6)	209 (74.9)		223 (59.6)	223 (82.0)	
	Total	367 (100.0)	279 (100.0)		374 (100.0)	272 (100.0)	

Pearson’s chi-square test

* p-value ≤ 0.05

**Caption**: n = number of individuals, which varies because of missing data

Table 2 also presents the association analysis between academic difficulties and difficulties memorizing tasks and arrangements that were only heard, using Pearson's chi-square test. Its results revealed a statistical significance between difficulty memorizing tasks and arrangements only heard with the answer: has/had academic difficulties related to concentration (p ≤ 0.001), memory (p ≤ 0.001), and planning (p ≤ 0.001). Those who did not have difficulty memorizing tasks and arrangements only heard tended not to have academic difficulties related to learning (p ≤ 0.001), understanding written information (p ≤ 0.001), understanding information that was not written and must be deduced (p ≤ 0.001), and finishing complex activities on time (p ≤ 0.001).

Table 3 shows the association analysis between auditory difficulties and academic difficulties related to concentration, using Pearson's chi-square test. It found a statistically significant association between difficulty concentrating and difficulty hearing and understanding in noisy environments (p ≤ 0.001) and memorizing tasks and arrangements that were only heard (p = 0.001). Furthermore, those who did not have academic difficulties related to concentration tended not to have difficulties in sound detection (p ≤ 0.001) and identification (p ≤ 0.001), selective and sustained attention to sounds (p ≤ 0.001), temporal sound perception (p ≤ 0.001), perception of different meanings due to changes in the tone of voice (p = 0.024), understanding sentences with double meanings (p = 0.007), understanding a message despite missing part of it (p = 0.001 ), and following tasks with varied stimuli (p = 0.001).

**Table 3:** Association analysis between academic difficulties in concentration, memory, and planning and auditory difficulties

Variables		Academic difficulties related to concentration			Academic difficulties related to memory			Academic difficulties related to planning		
		Yes n (%)	No n (%)	p-value	Yes n (%)	No n (%)	p-value	Yes n (%)	No n (%)	p-value
Problems detecting sounds	Yes	128 (31.4)	38 (16.0)	≤0.001*	113 (30.0)	53 (19.7)		100 (26.4)	66 (24.7)	
	No	280 (68.6)	200 (84.0)		264 (70.0)	216 (80.3)	0.003*	279 (73.6)	201 (75.3)	0.633
	Total	408 (100.0)	238 (100.0)		377 (100.0)	269 (100.0)		379 (100.0)	267 (100.0)	
Problems localizing and lateralizing sound sources	Yes	90 (22.1)	33 (13.9)	0.011*	87 (23.1)	36 (13.4)		76 (20.1)	47 (17.6)	
	No	318 (77.9)	205 (86.1)		290 (76.9)	233 (86.6)	0.002*	303 (79.9)	220 (82.4)	0.435
	Total	408 (100.0)	238 (100.0)		377 (100.0)	269 (100.0)		379 (100.0)	267 (100.0)	
Problems identifying sounds in general	Yes	69 (16.9)	13 (5.5)	≤0.001*	64 (17.0)	18 (6.7)		49 (12.9)	33 (12.4)	
	No	339 (83.1)	225 (94.5)		313 (83.0)	251 (93.3)	≤0.001*	330 (87.1)	234 (87.6)	0.831
	Total	408 (100.0)	238 (100.0)		377 (100.0)	269 (100.0)		379 (100.0)	267 (100.0)	
Problems discriminating sounds	Yes		26 (10.9)	0.818	49 (13.0)	24 (8.9)		41 (10.8)	32 (12.0)	
	No		212 (89.1)		328 (87.0)	245 (91.1)	0.107	338 (89.2)	235 (88.0)	0.645
	Total		238 (100.0)		377 (100.0)	269 (100.0)		379 (100.0)	267 (100.0)	
Problems with selective and sustained attention to sounds	Yes		74 (31.1)	≤0.001*	204 (54.1)	88 (32.7)		192 (50.7)	100 (37.5)	
	No		164 (68.9)		173 (45.9)	181 (67.3)	≤0.001*	187 (49.3)	167 (62.5)	0.001*
	Total		238 (100.0)		377 (100.0)	269 (100.0)		379 (100.0)	267 (100.0)	
Problems with short-term memory of sounds	Yes	232 (56.9)	66 (22.7)	≤0.001*	243 (64.5)	55 (20.4)		197 (52.0)	101 (37.8)	
	No	176 (43.1)	172 (72.3)		134 (35.5)	214 (79.6)	≤0.001*	182 (48.0)	166 (62.2)	≤0.001*
	Total	408 (100.0)	238 (100.0)		377 (100.0)	269 (100.0)		379 (100.0)	267 (100.0)	
Difficulties with temporal sound perception	Yes	209 (51.2)	81 (34.0)	≤0.001*	199 (52.8)	91 (33.8)		177 (46.7)	113 (42.3)	
	No	199 (48.8)	157 (66.0)		178 (47.2)	178 (66.2)	≤0.001*	202 (53.3)	154 (57.7)	0.270
	Total	408 (100.0)	238 (100.0)		377 (100.0)	269 (100.0)		379 (100.0)	267 (100.0)	
Difficulties hearing and understanding in noisy environments	Yes		114 (47.9)	≤0.001*	241 (63.9)	126 (46.8)		227 (59.9)	140 (52.4)	
	No		124 (52.1)		126 (36.1)	143 (53.2)	≤0.001*	152 (40.1)	127 (47.6)	0.060
	Total		238 (100.0)		377 (100.0)	269 (100.0)		379 (100.0)	267 (100.0)	
Difficulties perceiving different meanings according to changes in voice intonation	Yes	61 (15.0)	21 (8.8)	0.024*	56 (14.9)	26 (9.7)		60 (15.8)	22 (8.2)	
	No	347 (85.0)	217 (91.2)		321 (85.1)	243 (90.3)	0.054	319 (84.2)	245 (91.8)	0.004*
	Total	408 (100.0)	238 (100.0)		377 (100.0)	269 (100.0)		379 (100.0)	267 (100.0)	
Difficulties understanding jokes or words with double meaning	Yes	84 (20.6)	29 (12.2)	0.007*	76 (20.2)	37 (13.8)		78 (20.6)	35 (13.1)	
	No	324 (79.4)	209 (87.8)		301 (79.8)	232 (86.2)	0.035*	301 (79.4)	232 (86.9)	0.014*
	Total	408 (100.0)	238 (100.0)		377 (100.0)	269 (100.0)		379 (100.0)	267 (100.0)	
Difficulties perceiving and reproducing rhythms	Yes	121 (29.7)	58 (24.4)	0.148	118 (31.3)	61 (22.7)		118 (31.1)	61 (22.8)	
	No	287 (70.3)	180 (75.6)		259 (68.7)	208 (77.3)	0.016*	261 (68.9)	206 (77.2)	0.020*
	Total	408 (100.0)	238 (100.0)		377 (100.0)	269 (100.0)		379 (100.0)	267 (100.0)	
Difficulties understanding a message when part of it is not heard	Yes		43 (18.1)		140 (37.1)	45 (16.7)		126 (33.2)	59 (22.1)	
	No		195 (81.9)	0.001*	237 (62.9)	224 (83.3)	0.001*	253 (66.8)	208 (77.9)	0.002*
	Total		238 (100.0)		377 (100.0)	269 (100.0)		379 (100.0)	267 (100.0)	
Difficulties memorizing tasks and arrangements that are only heard	Yes		109 (45.8)		262 (69.5)	112 (41.6)		245 (64.6)	129 (48.3)	
	No		129 (54.2)	0.001*	115 (30.5)	157 (58.4)	0.001*	134 (35.4)	138 (51.7)	0.001*
	Total		238 (100.0)		377 (100.0)	269 (100.0)		379 (100.0)	267 (100.0)	
Difficulties following tasks with varied stimuli	Yes	135 (33.1)	38 (16.0)		131 (34.7)	42 (15.6)		131 (34.6)	42 (15.7)	
	No	273 (66.9)	200 (84.0)	0.001*	246 (65.3)	227 (84.4)	0.001*	248 (65.4)	225 (84.3)	0.001*
	Total	409 (100.0)	238 (100.0)		377 (100.0)	269 (100.0)		379 (100.0)	267 (100.0)	

Pearson’s chi-square test

* p-value ≤ 0.05

**Caption:** n = number of individuals

The association between auditory difficulties and academic difficulties related to memory, using Pearson's Chi-square test (Table 3), revealed a statistically significant association between memory difficulties and difficulty hearing and understanding in noisy environments (p ≤ 0.001) and memorizing tasks and arrangements that were only heard (p = 0.001). Moreover, those who did not have academic difficulties related to memory tended not to have difficulties in sound detection (p ≤ 0.001), identification (p ≤ 0.001), localization, and lateralization (p = 0.002), selective and sustained attention to sounds (p ≤ 0.001), short-term memory for sounds (p ≤ 0.001), temporal sound perception (p ≤ 0.001), understanding sentences with double meaning (p = 0.035), perceiving and reproducing rhythms (p = 0.016), understanding a message despite missing part of it (p = 0.001), and following tasks with varied stimuli (p = 0.001).

Table 3 also shows the association analysis between auditory difficulties and academic difficulties related to planning, using Pearson's Chi-square test. Statistically significant associations were observed between academic difficulties related to planning with difficulty memorizing tasks and arrangements that were only heard (p = 0.001). Furthermore, those who did not have academic difficulties related to planning tended not to have difficulties in selective and sustained attention to sounds (p = 0.001), short-term memory for sounds (p ≤ 0.001), perceiving different meanings due to changes in the tone of voice (p = 0.004), understanding jokes or words with double meanings (p = 0.014), perceiving and reproducing rhythms (p = 0.020), understanding a message despite missing part of it (p = 0.002), and following tasks with varied stimuli (p = 0.001).

## DISCUSSION

This study aimed to understand the academic and auditory profile of university students and graduates, based on their respective academic and auditory complaints. Due to the large number of questions researched in the questionnaire, only those with statistical relevance will be discussed.

Changes in listening skills coexist with learning difficulties, including reading and writing^(11,15)^, and impact academic performance. This may be related to difficulties in understanding, discriminating, recognizing, and recalling information presented only auditorily^(16)^ and associated with difficulties in following complex verbal instructions and maintaining concentration on tasks presented only verbally^(16)^ – which can be seen in the results of the present study. Most people who had complaints related to the auditory domain also had complaints related to the academic domain, and most people who had academic complaints also had auditory complaints.

This study shows that executive functions of selective attention, working memory, and planning were reported as the most recurrent difficulties. This can be better explained by reflecting on the role of executive functions in the activities of daily living and, more specifically, in academic life. Executive functions encompass skills necessary to accomplish a goal or task^(17)^ and help retain information more effectively^(18)^. They also enable behavior management, decision-making, risk assessment, adaptation to changing environments, engagement and targeting of actions and goals, and so forth^(19-21)^. Thus, executive functions play an important role in different areas of life, including learning and school performance, functioning, and independence in activities of daily living^(19,22)^.

The advancement in school and education levels poses greater demands on students regarding the environment and their executive functions^(19)^. Higher education requires planning, organization, and time management skills in increasing amounts and complexity, which can cause academic difficulties in individuals in this environment, who need greater autonomy and self-direction in their efforts toward learning^(19)^. This is demonstrated in that the three skills mentioned (considering concentration as a synonym for attention) were associated with academic difficulty by most students in this research and were statistically significantly associated with several other complaints.

The most prevalent auditory complaints in this research are also related to executive functions because speech comprehension in noise requires focused attention, ignoring the competing noise that comes from different sources in the social environment^(23)^, thus using selective attention. Likewise, memorizing and recalling instructions that have only been heard requires auditory working memory.

The questionnaire items on academic difficulties due to memory and concentration generally address these skills, without specifying memory as working memory or selective attention. Therefore, it is worth reflecting on the relevance of these skills in general in academic life. Attention plays an important role in perception, language, and memory, being the most influential factor in learning^(24)^. Sustained attention keeps the student's attention throughout an activity, such as reading a text, even if there are distracting factors^(23)^, while memory enables the analysis and acquisition of new knowledge, subsequent application in other contexts, and the development of various activities^(24)^.

Difficulty memorizing tasks that were only heard, which is related to auditory memory, was also one of the most prevalent complaints in this study^(25)^. Changes in auditory memory can lead to inappropriate use of oral and written language, difficulty in understanding someone else's speech and, consequently, problems in school performance and integration with peers^(25)^. This skill plays great relevance in retaining and remembering learning and, therefore, it is important that it is stimulated in school environments and in students' daily lives^(25)^.

Lastly, the most prevalent auditory complaints in this study were associated with complaints related to the cognitive functions of memory, attention, and planning, just as the absence of auditory complaints was associated with the absence of complaints involving cognitive skills. This can be justified because some CAP skills depend on other cognitive functions, such as attention, memory, language, executive functions, and so on^(26,27)^.

A study^(27)^ observed that executive functions share cognitive mechanisms underlying auditory skills, especially in tests that investigate focused attention, face perception, oral language, and working and episodic-semantic memory. Furthermore, there is evidence that, although some brain regions are specific for auditory stimuli, sensory data processing is interdependent and integrated, based on cognitive domains, especially attention, memory, and linguistic representations^(27)^, which explains the associations in this study.

It is important to highlight that the results of this article apply to the study population, with limitations such as the use of a self-reported questionnaire. Therefore, it is suggested that further research associate the questionnaire results with the CAP test battery and validated protocols for assessing academic skills.

Moreover, a study^(28)^ demonstrated an association between behavioral aspects and motivation to learn. Therefore, these factors need to be considered in future research, as they may influence the answers.

Since this study aimed to describe the auditory and academic complaints of adult students and employees of a public university in general, it was decided not to use hearing losses of any type and degree and syndromic, neurological, and/or cognitive changes as exclusion criteria. However, future studies should consider these criteria, as these conditions may interfere with the complaints and results. They should also control the age, investigating individuals by age group.

As contributions, the study presented statistically significant associations between cognitive functions and self-reported auditory skills, which add to previous research and support future studies. The research also favors the development of educational and intervention planning by demonstrating the relevance of executive functions in academic life, especially in higher education, which must be considered for better student achievement and a better therapeutic prognosis.

Therefore, these factors should be considered in CAP assessments; in auditory interventions, ensuring the presence or absence of associated cognitive factors; and in academic life, to provide students with an adequate listening environment for learning and identifying possible gaps in cognitive development that must be addressed to enable the full acquisition of knowledge.

## CONCLUSION

The results show hearing and academic difficulties among university and graduate adults, among which the most prevalent complaints were academic difficulties related to concentration, memory, and planning, and auditory difficulties in understanding speech in noise and memorizing tasks that were only heard.

It was also observed that adults' auditory and academic complaints are associated, marked by the relationship between cognitive and auditory aspects.
