# Supplementary material for: Auditory and academic skills self-perception in adults
Source: Codas. 2024 Jun 14;36(3):e20230098. doi: 10.1590/2317-1782/20242023098en (PMC466998; doi:10.1590/2317-1782/20242023098en)
Supplement: Translation

# Translation: Autopercepção sobre habilidades auditivas e acadêmicas de adultos

**DOI:** 10.1590/2317-1782/20242023098pt

**Authors:** Bruna Stéfanie Pereira, Luciana Macedo de Resende, Luciana Cássia de Jesus, Andrezza Gonzalez Escarce, Luciana Mendonça Alves

**Affiliations:** 1 Departamento de Fonoaudiologia, Faculdade de Medicina, Universidade Federal de Minas Gerais – UFMG - Belo Horizonte (MG), Brasil.

**Keywords:** Percepção Auditiva, Aprendizagem, Audição, Fonoaudiologia, Estudantes, Autorrelato, Adulto

## Abstract

**Objetivo:**

Descrever e analisar queixas auditivas e acadêmicas de universitários e funcionários de uma universidade pública federal.

**Métodos:**

O estudo foi realizado por amostra não-probabilística. A Escala de Autopercepção de Habilidades do Processamento Auditivo Central com adaptações foi utilizada para cumprir os objetivos da pesquisa. Esta possui 14 questões sobre queixas relacionadas às habilidades auditivas e 12 relacionadas ao ambiente acadêmico. Foi realizada a análise descritiva dos dados por meio da distribuição de frequência das variáveis categóricas e, para as análises de associação, foi utilizado o teste Qui-quadrado de Pearson.

**Resultados:**

Participaram da pesquisa 646 indivíduos com faixa etária entre 17 e 67 anos. As queixas mais prevalentes foram: dificuldade acadêmica relacionada à memória, concentração e planejamento, ouvir e compreender a fala no ruído, e memorização de tarefas que foram apenas ouvidas. Houve associação com significância estatística bidirecional entre as queixas acadêmicas e auditivas.

**Conclusão:**

Foi possível observar que há associação entre queixas auditivas e acadêmicas em adultos, marcada pela relação de aspectos cognitivos com aspectos auditivos. É relevante que esses fatores sejam considerados ao realizar avaliações do Processamento Auditivo Central, ao se intervir em pacientes com queixas auditivas, e na vida estudantil.

## INTRODUÇÃO

A audição permite aos indivíduos estabelecerem contato com o meio em que estão inseridos, mas, além disso, tem grande relevância no desenvolvimento da aprendizagem, pois possibilita analisar e compreender a informação auditiva em diversos contextos^(1)^.

O Processamento Auditivo Central (PAC) é composto por um conjunto de habilidades como: identificar a lateralização e a localização espacial do som; compreender a fala no ruído; compreender uma mensagem distorcida e fragmentada; direcionar a atenção a estímulos apresentados em uma orelha, em detrimento de sons apresentados à orelha oposta; reconhecer diferentes sons apresentados a ambas orelhas simultaneamente; discriminar pequenas mudanças nos estímulos sonoros; e detectar e perceber modulações e intervalos mínimos em uma sequência sonora^(2)^.

Dessa forma, as habilidades auditivas centrais permitem que palavras faladas, músicas, ruídos e sons do ambiente sejam reconhecidos; os fonemas surdos e sonoros da língua materna sejam diferenciados; fonemas semelhantes sejam reconhecidos; e os sons ouvidos sejam integrados e ordenados para registrar uma informação auditiva^(3)^. Ademais, auxilia na organização fonológica, sintática e na prosódia^(3)^.

Além dessas habilidades, atenção, memória e compreensão auditivas são outras competências imprescindíveis para o processamento dos estímulos sonoros. A atenção auditiva tem papel importante na recepção e expressão da linguagem^(3)^, enquanto a memória auditiva auxilia na retenção das informações auditivas^(4)^. Por fim, a compreensão auditiva permite entender o significado da informação auditiva associada às outras habilidades auditivas citadas^(5)^.

Assim, fica evidente a relação das habilidades auditivas com o processo de aprendizagem, visto que para aprender é necessário discriminar, reconhecer, reter e conferir sentidos e definições aos sons, além de integrar a informação auditiva aos outros sentidos para compreender aspectos melódicos da fala, informações com duplo sentido ou que precisam ser inferidas^(6,7)^.

O PAC pode ser avaliado de forma comportamental, por meio de uma bateria de testes importantes para analisar as habilidades auditivas funcionalmente^(2)^, direcionando os processos terapêuticos, e por meio de procedimentos eletrofisiológicos^(2)^. Todavia, a literatura tem mencionado a utilização de questionários, de forma complementar, que possibilitam a identificação de sintomas e sinais de risco para o Transtorno do Processamento Auditivo Central (TPAC)^(8)^. Esses instrumentos, além de serem de baixo custo e de preenchimento simples, permitem a coleta de informações relevantes sobre o comportamento auditivo no ambiente naturalístico, visto que tais dados não são possíveis de serem verificados pelos profissionais em ambientes diferentes do consultório^(8-10)^.

Devido à escassez de instrumentos de rastreio das habilidades do PAC direcionados a população brasileira adulta^(11)^, foi elaborada a Escala de Autopercepção de Habilidades do Processamento Auditivo Central (EAPAC) ^(11)^ para adultos jovens. Esta escala tem o intuito de identificar habilidades auditivas que apresentam risco de alteração e possibilitar o desenvolvimento de estratégias para promoção de saúde auditiva e de aprendizagem^(10)^ nessa população. A escala é composta por questões relacionadas às queixas auditivas e de aprendizagem. Uma pesquisa^(11)^ buscou validar essa escala e pode-se concluir, por meio da análise psicométrica, que a EAPAC apresentou boa correlação entre seus itens e resultados válidos, demonstrando confiabilidade e possibilitando a realização de inferências sobre possíveis alterações no Processamento Auditivo.

A partir do contexto apresentado, levanta-se as seguintes questões: quais seriam as queixas auditivas e acadêmicas mais prevalentes em universitários e ex-universitários? Existiria associação entre queixas auditivas e acadêmicas neste grupo? Há, na literatura, estudos que abordam a influência do Processamento Auditivo nas dificuldades de aprendizagem em crianças e apresentam correlações positivas entre os dois aspectos^(12-14)^. Entretanto, ainda existem lacunas acerca do tema com o público-alvo adulto.

Dessa forma, o presente estudo teve por objetivo descrever e analisar queixas auditivas e acadêmicas de universitários, obtidas por meio das respostas desse grupo ao questionário Escala de Autopercepção das Habilidades do Processamento Auditivo – EAPAC^(11)^ com adaptações realizadas pelos autores.

## MÉTODO

Trata-se de um estudo transversal descritivo, comparativo, que foi aprovado pelo Comitê de Ética em Pesquisa (COEP) da Universidade Federal de Minas Gerais (UFMG) sob parecer nº 5.137.573. Todos os participantes foram informados quanto aos objetivos e procedimentos do estudo e assinaram o Termo de Consentimento Livre e Esclarecido (TCLE).

Foram convidados a participar da pesquisa estudantes e funcionários de uma universidade pública federal, por meio de correio eletrônico enviado pela Diretoria de Tecnologia da Informação da Universidade. O convite foi enviado para o *e-mail* institucional de cerca de 50.561 indivíduos, com um *link* para o programa *Google Forms* que continha o TCLE e o questionário EAPAC^(11)^ com adaptações realizadas pelos autores. Foram inseridas sete perguntas relacionadas ao domínio auditivo e cinco relacionadas ao domínio acadêmico, por apresentarem possível associação às queixas auditivas.

A EAPAC^(11)^ com adaptações (Quadro 1) é composta por 26 questões, sendo 25 com opções de respostas sobre a dificuldade apresentada e uma questão discursiva. A Escala possui 14 questões sobre queixas relacionadas às habilidades auditivas (detecção, localização e lateralização da fonte sonora; reconhecimento, discriminação e atenção seletiva e sustentada; memória auditiva de curta duração e aspectos temporais da audição) e 12 relacionadas ao ambiente acadêmico (concentração, memória, planejamento e aprendizagem).

**Quadro 1:** Escala de Autopercepção das Habilidades do Processamento Auditivo – EAPAC com adaptações

EAPAC - ESCALA DE AUTOPERCEPÇÃO DAS HABILIDADES DO PROCESSAMENTO AUDITIVO EM ADULTOS: EXPANDIDA		RESPOSTAS	
Nome:__________________________________________ Sexo: __________________ Idade:____________________ Escolaridade:_______________________________________________________ Telefone: ( ) ___________ - ____________ Data:_____/_____/_________ **Você fez o ensino médio em ( ) escola pública ( ) escola particular ( ) não se aplica**		**Sim**	Não
Domínio auditivo Domínio acadêmico		PONTUAÇÃO	
QUESTÕES		**(1)**	(0)
**Q1**	Você acredita ter problemas na detecção do som (som em geral, fala ou outros sons)?		
**Q2**	Você acredita ter problemas na localização e lateralização da fonte sonora (saber de onde chamam quando à distância, por exemplo)?		
**Q3**	Você acredita ter problemas na identificação dos sons em geral?		
**Q4**	Você acredita ter problemas na discriminação do som (diferenciar sons da fala, por exemplo ouvir S e Z)?		
**Q5**	Você acredita ter problemas na atenção seletiva e sustentada ao som (ouvir e entender a fala do professor, mesmo que haja outras conversas na sala ou ruído externo, por exemplo)?		
**Q6**	Você acredita ter problemas de memória de curta duração relacionados ao som (lembrar-se de coisas que apenas ouviu, como textos curtos, uma aula, por exemplo)?		
**Q7**	Você acredita ter dificuldades para perceber os sons no tempo? Por exemplo, entender alguém que fala muito rápido ou que articula as palavras sem clareza.		
**Q8**	Você acredita ter dificuldades para ouvir e entender a fala em situações ruidosas? Por exemplo, conversando no ponto de ônibus, em restaurantes etc.		
**Q9**	Você apresenta ou já apresentou dificuldades acadêmicas relacionadas à concentração em algum momento da sua vida acadêmica ou durante a atividade profissional?		
**Q10**	Você apresenta ou já apresentou dificuldades acadêmicas relacionadas à memória em algum momento da sua vida acadêmica ou durante a atividade profissional?		
**Q11**	Você apresenta ou já apresentou dificuldades acadêmicas relacionadas a planejamento em algum momento da sua vida acadêmica ou durante a atividade profissional?		
**Q12**	Você apresenta ou já apresentou dificuldades acadêmicas relacionadas à aprendizagem em algum momento da sua vida acadêmica ou durante a formação profissional?		
**Q13**	Você apresenta dificuldade para compreender informações lidas?		
**Q14**	Você apresenta dificuldade para compreender as informações que não foram escritas no texto pelo autor e que precisam ser deduzidas (que estão nas entrelinhas)?		
**Q15**	Você apresenta trocas de letras que representam sons semelhantes, na escrita ou leitura? A seguir são apresentadas algumas letras que representam sons semelhantes: B - P, D – T, G - C/Q/K, V – F, Z – S, J – X/CH. Exemplo: Cola – Gola, Já – Chá, Vaca – Faca		
**Q16**	Você apresenta dificuldade para fazer e perceber as pausas no texto, devido os sinais de pontuação?		
**Q17**	Você é fluente em alguma língua estrangeira?		
**Q18**	Você estuda ou estudou alguma língua estrangeira? Se sim, qual e por quanto tempo? Caso não estude ou não estudou, responda não.		
**Q19**	Você apresenta dificuldade para aprender uma nova língua?		
**Q20**	Você apresenta dificuldade para perceber quando a pessoa quer dar um significado diferente à informação dita realizando modificação no seu tom de voz?		
**Q21**	Você apresenta dificuldade para entender piadas ou palavras com duplo sentido?		
**Q22**	Você apresenta dificuldade para perceber e reproduzir ritmos?		
**Q23**	Se você estiver conversando com alguém e não ouvir um trecho que a pessoa disse, tem dificuldade de compreender toda a mensagem/fala do outro?		
**Q24**	Você apresenta dificuldade em memorizar tarefas e combinados que foram apenas ouvidas (sem realizar anotações)?		
**Q25**	Você apresenta dificuldade em finalizar atividades complexas (que exigem formulação e execução de respostas) no prazo estipulado?		
**Q26**	Você apresenta dificuldade em acompanhar tarefas com estímulos variados, como sons, imagens, textos e animações?		

Foram também coletadas informações quanto às características sociodemográficas (nome, idade, sexo, naturalidade, escolaridade e modalidade da escola em que cursou o Ensino Médio).

O estudo foi realizado por amostra não-probabilística. A amostra inicial foi composta por 697 indivíduos inseridos na universidade pesquisada, que responderam ao questionário Escala de Autopercepção das Habilidades do Processamento Auditivo – EAPAC^(11)^ com adaptações.

Adotou-se como critério de inclusão ser associado à Universidade como estudante ou servidor e, como critério de exclusão, não apresentar, ao menos, nível de escolaridade de Ensino Superior incompleto, pois desejava-se caracterizar as dificuldades acadêmicas e auditivas de indivíduos que experienciaram a Universidade. Desta forma, 51 participantes foram excluídos do estudo, pois havia cursado apenas o Ensino Médio, totalizando uma amostra com 646 integrantes.

Os participantes foram divididos em quatro grupos, sendo eles: com queixas auditivas e acadêmicas composto por 538 pessoas, com queixas acadêmicas formado por 39 indivíduos, com queixas auditivas composto por 45 participantes e sem queixas formado por 24 indivíduos.

Foi realizada a análise descritiva dos dados, por meio da distribuição de frequência das variáveis categóricas.

Para as análises de associação foi utilizado o teste Qui-quadrado de Pearson, sendo consideradas como associações estatisticamente significantes as que apresentaram valor de p ≤ 0,05.

Para entrada, processamento e análise dos dados foi utilizado o software SPSS versão 25.0, após a inserção dos dados em planilha no *Excel* ^®^.

## RESULTADOS

A Tabela 1 apresenta a descrição das características gerais dos participantes do estudo. Um participante obteve o título de escolaridade de Ensino Médio por meio de prova autorizada para tal e, por isso, não se aplica a questão de modalidade da escola que cursou o Ensino Médio para este estudante.

**Tabela 1:** Análise descritiva das características gerais da amostra

Dados	Variáveis	Valores
Idade	Mínimo	17
	Máximo	67
	Mediana	29
	Abaixo de 30 anos n(%)	328(50,8)
	30 anos ou mais n(%)	318(49,2)
Sexo	Masculino n(%)	208(32,2)
	Feminino n(%)	438(67,8)
Naturalidade	Brasileiro n(%)	645(99,8)
	Estrangeiro n(%)	1(0,2)
Escolaridade	Superior incompleto n(%)	292(45,2)
	Superior completo n(%)	110(17,0)
	Pós-graduação n(%)	86(13,3)
	Mestrado n(%)	120(18,6)
	Doutorado n(%)	38(5,9)
Ensino médio	Escola pública n(%)	400(61,9)
	Escola particular n(%)	245(37,9)
	Não se aplica n(%)	1(0,2)
Queixas	Sem queixa n(%)	24(3,7)
	Queixa auditiva e acadêmica n(%)	538(83,3)
	Queixa auditiva n(%)	45(7,0)
	Queixa acadêmica n(%)	39(6,0)

**Legenda:** n=número de participantes

Os participantes da pesquisa eram, em sua maioria, do sexo feminino (67,8%), com faixa etária entre 17 e 67 anos, naturalidade brasileira (99,8%), Ensino Superior Incompleto (45,2%), presença concomitante de queixas auditivas e acadêmicas (83,3%) e egressos de escola pública no Ensino Médio (61,9%).

Na Figura 1, é possível observar a prevalência de queixas auditivas e acadêmicas relatadas no Questionário. As queixas mais prevalentes foram: dificuldade acadêmica relacionada à concentração (63,16%), planejamento (58,67%) e memória (58,36%), memorização de tarefas que foram apenas ouvidas (57,9%), e ouvir e compreender a fala no ruído (56,8%).

**Figura 1:** Gráfico da prevalência de queixas auditivas e extra auditivas apresentadas no questionário

A partir das queixas mais prevalentes relatadas, foi realizada a análise de associação entre elas e as demais queixas. As queixas de dificuldades acadêmicas relacionadas a concentração, memória e planejamento foram associadas às questões de domínio auditivo, enquanto as queixas de dificuldades auditivas relacionadas a ouvir e entender em ambientes ruidosos e dificuldade em memorizar tarefas e combinados apresentados apenas de forma oral foram associadas às questões de domínio acadêmico. Tal divisão foi realizada para verificar a existência de associação entre queixas auditivas e acadêmicas dos participantes.

Na Tabela 2, destacam-se as associações entre dificuldade para ouvir e entender em ambientes ruidosos e dificuldades acadêmicas, por meio do teste Qui-quadrado de *Pearson*. Em sua análise foi possível observar que houve associação com significância estatística entre a dificuldade para ouvir e entender em ambientes ruidosos com apresentar ou ter apresentado dificuldades acadêmicas relacionadas à memória (p≤0,001). Além disso, foi observada tendência de quem não apresenta dificuldade em ambientes ruidosos também não apresentar dificuldades de aprendizagem (p=0,027), de compreender informações lidas (p=0,008), de compreender informações que precisam ser deduzidas (p=0,028), e de realizar tarefas complexas no prazo (p=0,005).

**Tabela 2:** Associação entre dificuldade de compreender no ruído, memorizar tarefas ouvidas e dificuldades acadêmicas

Variáveis		Dificuldade com ambientes ruidosos			Dificuldade em memorizar tarefas ouvidas		
		Sim	Não	valor-p	Sim	Não	valor-p
		n (%)	n (%)		n (%)	n (%)	
Dificuldades acadêmicas relacionadas à concentração	Sim	241 (65,7)	136 (48,7)	≤0,001*	265 (70,9)	143 (52,6)	≤0,001*
	Não	126 (34,3)	153 (51,3)		109 (29,1)	129 (47,4)	
	Total	367 (100,0)	279 (100,0)		374 (100,0)	272 (100,0)	
Dificuldades acadêmicas relacionadas a memória	Sim	241 (65,7)	136 (48,7)	≤0,001*	262 (70,1)	115 (42,3)	≤0,001*
	Não	126 (34,3)	153 (51,3)		112 (29,9)	157 (57,7)	
	Total	367 (100,0)	279 (100,0)		374 (100,0)	272 (100,0)	
Dificuldades acadêmicas relacionadas ao planejamento	Sim	227 (61,9)	152 (54,5)	0,059	245 (65,5)	134 (49,3)	≤0,001*
	Não	140 (38,1)	127 (45,5)		129 (34,5)	138 (50,7)	
	Total	367 (100,0)	279 (100,0)		374 (100,0)	272 (100,0)	
Dificuldades acadêmicas relacionadas a aprendizagem	Sim	186 (50,7)	117 (41,9)	0,027*	205 (54,8)	98 (36,0)	≤0,001*
	Não	181 (49,3)	162 (58,1)		169 (45,2)	174 (64,0)	
	Total	367 (100,0)	279 (100,0)		374 (100,0)	272 (100,0)	
Dificuldade para compreender informações lidas	Sim	67 (18,3)	30 (10,8)	0,008*	72 (19,3)	25 (9,2)	≤0,001*
	Não	300 (81,7)	249 (89.2)		302 (80,7)	247 (90,8)	
	Total	367 (100,0)	279 (100,0)		374 (100,0)	272 (100,0)	
Dificuldade para compreender informações não escritas/deduzidas	Sim	104 (28,3)	58 (20,8)	0,028*	120 (32,1)	42 (15,4)	≤0,001*
	Não	263 (71,7)	221 (79,2)		254 (67,9	230 (84,6)	
	Total	368 (100,0)	279 (100,0)		374 (100,0)	272 (100,0)	
Trocas de letras com sons semelhantes, na escrita ou leitura	Sim	52 (14,2)	32 (11,5)	0,312	54 (14,4)	30 (11,0)	0,203
	Não	315 (85,8)	247 (88,5)		320 (85,6)	242 (89,0)	
	Total	367 (100,0)	279 (100,0)		374 (100,0)	272 (100,0)	
Dificuldade para fazer/perceber pausas no texto devido à pontuação	Sim	44 (12,0)	22 (7,9)	0,088	45 (12,0)	21 (7,7)	0,074
	Não	323 (88,0)	257 (92,1)		329 (88,0)	251 (92,3)	
	Total	367 (100,0)	279 (100,0)		374 (100,0)	272 (100,0)	
Dificuldade para aprender nova língua	Sim	141 (42,3)	96 (38,1)	0,300	151 (43,5)	86 (36,1)	0,075
	Não	192 (57,7)	156 (61,9)		196 (56,5)	152 (63,9)	
	Total	333 (100,0)	252 (100,0)		374 (100,0)	272 (100,0)	
Dificuldade em finalizar atividades complexas	Sim	130 (35,4)	70 (25,1)	0,005*	151 (40,4)	49 (18,0)	≤0,001*
	Não	237 (64,6)	209 (74,9)		223 (59,6)	223 (82,0)	
	Total	367 (100,0)	279 (100,0)		374 (100,0)	272 (100,0)	

Teste Qui-quadrado de Pearson

* valor de p≤0,05

**Legenda:** n= número de indivíduos, varia devido a dados faltantes

A análise de associação entre dificuldades memorizar tarefas e combinados que foram apenas ouvidos e dificuldades acadêmicas, por meio do teste do teste Qui-quadrado de *Pearson*, destaca-se também na Tabela 2. Seus resultados revelaram que houve significância estatística entre dificuldade em memorizar tarefas e combinados apenas ouvidos com a resposta: apresenta(ou) dificuldades acadêmicas relacionadas à concentração (p≤0,001), memória (p≤0,001) e planejamento (p≤0,001). Quem não apresentou dificuldade em memorizar tarefas e combinados apenas ouvidos, apresentou tendência em não apresentar dificuldades acadêmicas relacionadas à aprendizagem (p≤0,001), para compreender informações lidas (p≤0,001), para compreender informações que não foram escritas e precisam ser deduzidas (p≤0,001), e em finalizar atividades complexas no prazo (p≤0,001).

A análise de associação entre dificuldades acadêmicas relacionadas a concentração e dificuldades auditivas, por meio do teste do teste Qui-quadrado de *Pearson*, é apresentada na Tabela 3. Em sua análise foi possível observar que houve associação com significância estatística entre a dificuldade de concentração com: dificuldade para ouvir e entender em ambientes ruidosos (p≤0,001) e dificuldade em memorizar tarefas e combinados que foram apenas ouvidos (p=0,001). No mais, quem não apresentou dificuldade acadêmica relacionada a concentração tendeu a não apresentar dificuldade na detecção (p≤0,001) e identificação sonora (p≤0,001), na atenção seletiva e sustentada de sons (p≤0,001), na percepção de sons no tempo (p≤0,001), em perceber significados diferentes devido à modificação no tom de voz (p=0,024), na compreensão de frases com duplo sentido (p=0,007), em compreender a mensagem se não ouviu um trecho (p=0,001), e em acompanhar tarefas com estímulos variados (p=0,001).

**Tabela 3:** Análise de associação entre dificuldades acadêmicas de concentração, memória e planejamento e dificuldades auditivas

Variáveis		Dificuldades acadêmicas relacionadas a concentração			Dificuldades acadêmicas relacionadas a memória				Dificuldades acadêmicas relacionadas ao planejamento			
		**Sim** **n (%)**	**Não** **n (%)**	**valor-p**	**Sim** **n (%)**	**Não** **n (%)**		**valor-p**	**Sim** **n (%)**	**Não** **n (%)**	**Valor-p**	
Problemas na detecção do som	Sim	128 (31,4)	38 (16,0)	≤0,001*	113 (30,0)		53 (19,7)		100 (26,4)	66 (24,7)		
	Não	280 (68,6)	200 (84,0)		264 (70,0)		216 (80,3)	0,003*	279 (73,6)	201 (75,3)		0,633
	Total	408 (100,0)	238 (100,0)		377 (100,0)		269 (100,0)		379 (100,0)	267 (100,0)		
Problemas de localização e lateralização da fonte sonora	Sim	90 (22,1)	33 (13,9)	0,011*	87 (23,1)		36 (13,4)		76 (20,1)	47 (17,6)		
	Não	318 (77,9)	205 (86,1)		290 (76,9)		233 (86,6)	0,002*	303 (79,9)	220 (82,4)		0,435
	Total	408 (100,0)	238 (100,0)		377 (100,0)		269 (100,0)		379 (100,0)	267 (100,0)		
Problemas na identificação de sons em geral	Sim	69 (16,9)	13 (5,5)	≤0,001*	64 (17,0)		18 (6,7)		49 (12,9)	33 (12,4)		
	Não	339 (83,1)	225 (94,5)		313 (83,0)		251 (93,3)	≤0,001*	330 (87,1)	234 (87,6)		0,831
	Total	408 (100,0)	238 (100,0)		377 (100,0)		269 (100,0)		379 (100,0)	267 (100,0)		
Problemas na discriminação do som	Sim		26 (10,9)	0,818	49 (13,0)		24 (8,9)		41 (10,8)	32 (12,0)		
	Não		212 (89,1)		328 (87,0)		245 (91,1)	0,107	338 (89,2)	235 (88,0)		0,645
	Total		238 (100,0)		377 (100,0)		269 (100,0)		379 (100,0)	267 (100,0)		
Problemas na atenção seletiva e sustentada de sons	Sim		74 (31,1)	≤0,001*	204 (54,1)		88 (32,7)		192 (50,7)	100 (37,5)		
	Não		164 (68,9)		173 (45,9)		181 (67,3)	≤0,001*	187 (49,3)	167 (62,5)		0,001*
	Total		238 (100,0)		377 (100,0)		269 (100,0)		379 (100,0)	267 (100,0)		
Problemas de memória de curta duração de sons	Sim	232 (56,9)	66 (22,7)	≤0,001*	243 (64,5)		55 (20,4)		197 (52,0)	101 (37,8)		
	Não	176 (43,1)	172 (72,3)		134 (35,5)		214 (79,6)	≤0,001*	182 (48,0)	166 (62,2)		≤0,001*
	Total	408 (100,0)	238 (100,0)		377 (100,0)		269 (100,0)		379 (100,0)	267 (100,0)		
Dificuldade para perceber os sons no tempo	Sim	209 (51,2)	81 (34,0)	≤0,001*	199 (52,8)		91 (33,8)		177 (46,7)	113 (42,3)		
	Não	199 (48,8)	157 (66,0)		178 (47,2)		178 (66,2)	≤0,001*	202 (53,3)	154 (57,7)		0,270
	Total	408 (100,0)	238 (100,0)		377 (100,0)		269 (100,0)		379 (100,0)	267 (100,0)		
Dificuldade para ouvir e entender em ambientes ruidosos	Sim		114 (47,9)	≤0,001*	241 (63,9)		126 (46,8)		227 (59,9)	140 (52,4)		
	Não		124 (52,1)		126 (36,1)		143 (53,2)	≤0,001*	152 (40,1)	127 (47,6)		0,060
	Total		238 (100,0)		377 (100,0)		269 (100,0)		379 (100,0)	267 (100,0)		
Dificuldade para perceber significados diferentes devido a mudança no tom de voz	Sim	61 (15,0)	21 (8,8)	0,024*	56 (14,9)		26 (9,7)		60 (15,8)	22 (8,2)		
	Não	347 (85,0)	217 (91,2)		321 (85,1)		243 (90,3)	0,054	319 (84,2)	245 (91,8)		0,004*
	Total	408 (100,0)	238 (100,0)		377 (100,0)		269 (100,0)		379 (100,0)	267 (100,0)		
Dificuldades para entender piadas ou palavras com duplo sentido	Sim	84 (20,6)	29 (12,2)	0,007*	76 (20,2)		37 (13,8)		78 (20,6)	35 (13,1)		
	Não	324 (79,4)	209 (87,8)		301 (79,8)		232 (86,2)	0,035*	301 (79,4)	232 (86,9)		0,014*
	Total	408 (100,0)	238 (100,0)		377 (100,0)		269 (100,0)		379 (100,0)	267 (100,0)		
Dificuldade para perceber e reproduzir ritmos	Sim	121 (29,7)	58 (24,4)	0,148	118 (31,3)		61 (22,7)		118 (31,1)	61 (22,8)		
	Não	287 (70,3)	180 (75,6)		259 (68,7)		208 (77,3)	0,016*	261 (68,9)	206 (77,2)		0,020*
	Total	408 (100,0)	238 (100,0)		377 (100,0)		269 (100,0)		379 (100,0)	267 (100,0)		
Dificuldade em compreender a mensagem se não ouviu um trecho	Sim		43 (18,1)		140 (37,1)		45 (16,7)		126 (33,2)	59 (22,1)		
	Não		195 (81,9)	0,001*	237 (62,9)		224 (83,3)	0,001*	253 (66,8)	208 (77,9)		0,002*
	Total		238 (100,0)		377 (100,0)		269 (100,0)		379 (100,0)	267 (100,0)		
Dificuldade em memorizar tarefas e combinados apenas ouvidos	Sim		109 (45,8)		262 (69,5)		112 (41,6)		245 (64,6)	129 (48,3)		
	Não		129 (54,2)	0,001*	115 (30,5)		157 (58,4)	0,001*	134 (35,4)	138 (51,7)		0,001*
	Total		238 (100,0)		377 (100,0)		269 (100,0)		379 (100,0)	267 (100,0)		
Dificuldade em acompanhar tarefas com estímulos variados	Sim	135 (33,1)	38 (16,0)		131 (34,7)		42 (15,6)		131 (34,6)	42 (15,7)		
	Não	273 (66,9)	200 (84,0)	0,001*	246 (65,3)		227 (84,4)	0,001*	248 (65,4)	225 (84,3)		0,001*
	Total	409 (100,0)	238 (100,0)		377 (100,0)		269 (100,0)		379 (100,0)	267 (100,0)		

Teste Qui-quadrado de Pearson

* valor de p≤0,05

**Legenda:** n = número de indivíduos

A associação entre dificuldades acadêmicas relacionadas à memória e dificuldades auditivas, por meio do teste do teste Qui-quadrado de *Pearson* (Tabela 3), revelou que houve associação com significância estatística entre a dificuldade de memória com: dificuldade para ouvir e entender em ambientes ruidosos (p≤0,001) e dificuldade em memorizar tarefas e combinados que foram apenas ouvidos (p=0,001). Ademais, quem não apresentou dificuldades acadêmicas relacionadas à memória apresentou tendência a não apresentar dificuldades na detecção (p≤0,001), identificação (p≤0,001), localização e lateralização sonora (p=0,002), na atenção seletiva e sustentada de sons (p≤0,001), na memória de curta duração aos sons (p≤0,001), para perceber sons no tempo (p≤0,001), para entender frases com duplo sentido (p=0,035), para perceber e reproduzir ritmos (p=0,016), em compreender a mensagem se não ouviu um trecho (p=0,001), e em acompanhar tarefas com estímulos variados (p=0,001).

Na Tabela 3 consta, ainda, a análise de associação entre dificuldades acadêmicas relacionadas a planejamento e dificuldades auditivas, por meio do teste do teste Qui-quadrado de *Pearson*. Observa-se associações com significância estatística entre dificuldades acadêmicas relacionadas a planejamento com dificuldade em memorizar tarefas e combinados que foram apenas ouvidos (p=0,001). Outrossim, quem não apresentou dificuldade acadêmica relacionada ao planejamento, tendeu a não apresentar dificuldades na atenção seletiva e sustentada de sons (p=0,001), na memória de curta duração aos sons (p≤0,001), para perceber significados diferentes devido à modificação no tom de voz (p=0,004), para entender piadas ou palavras com duplo sentido (p=0,014), em perceber e reproduzir ritmos (p=0,020), em compreender a mensagem se não ouviu um trecho (p=0,002), e em acompanhar tarefas com estímulos variados (p=0,001).

## DISCUSSÃO

No presente estudo objetivou-se conhecer o perfil acadêmico e auditivo de universitários e graduados, a partir das respectivas queixas acadêmicas e auditivas. Devido ao grande número de questões pesquisadas no Questionário, serão discutidos apenas aqueles que obtiveram relevância estatística.

Alterações nas habilidades auditivas coexistem com dificuldades de aprendizagem, incluindo dificuldades na leitura e escrita^(11,15)^, e impactam no desempenho acadêmico, podendo estar relacionadas às dificuldades de compreender, discriminar, reconhecer e evocar informações apresentadas apenas auditivamente^(16)^. Estão associadas, ainda, às dificuldades de seguir instruções verbais complexas e de manter a concentração em tarefas apresentadas apenas verbalmente^(16)^. Isso pode ser observado nos resultados encontrados no presente estudo, visto que a maioria das pessoas que apresentaram queixas relacionadas ao domínio auditivo, apresentaram também queixas relacionadas ao domínio acadêmico e, a maior parte das pessoas que apresentaram queixas acadêmicas, apresentaram também queixas auditivas.

Percebe-se que, neste estudo, as funções executivas (FE) de atenção seletiva, memória de trabalho e planejamento foram relatadas como dificuldades mais preponderantemente. Isto pode ser mais bem explicado ao se refletir sobre o papel das FE nas atividades de vida diária e, mais especificamente, na vida acadêmica. As funções executivas são compostas por habilidades necessárias para a realização de um objetivo ou tarefa^(17)^, e fornecem suporte para uma retenção mais efetiva das informações^(18)^. Além disso, elas permitem o gerenciamento de comportamentos, tomada de decisões, avaliação de riscos, adaptação de ambientes em mudança, engajamento e direcionamento de ações e metas, entre outros^(19-21)^. Por isso, as funções executivas possuem importante papel em diferentes áreas da vida, incluindo aprendizagem e desempenho escolar, funcionalidade e independência em atividades da vida diária^(19,22)^.

Com o avanço dos níveis escolares e do grau de escolaridade, há um aumento das demandas ambientais e da exigência sobre as funções executivas dos estudantes^(19)^. As habilidades de planejamento, organização e manejo de tempo são demandadas de maneira progressiva e em maior complexidade no Ensino Superior, o que pode causar dificuldades acadêmicas nos indivíduos deste meio, que necessitam de maior autonomia e autodirecionamento dos esforços para a aprendizagem^(19)^. Isto se torna evidente ao notar que as três habilidades citadas, considerando a concentração como sinônimo de atenção, foram associadas à dificuldade acadêmica pela maior parte dos estudantes da presente pesquisa e obtiveram associação estatisticamente significante entre várias das outras queixas expostas.

As queixas mais prevalentes relacionadas ao domínio auditivo encontradas na atual pesquisa também apresentam relação com as funções executivas. Isto pois, para realizar a tarefa de compreender no ruído, é necessário focalizar sua atenção a este e ignorar o ruído competitivo, proveniente de diversas fontes do ambiente social^(23)^, fazendo uso, assim, da habilidade de atenção seletiva. Da mesma maneira, para memorizar e recordar instruções que foram apenas ouvidas é preciso utilizar a memória de trabalho auditiva.

As questões assinaladas no Questionário relacionadas à dificuldade acadêmica devido à memória e concentração diziam respeito a essas habilidades em geral, sem especificar a memória como a de trabalho e a atenção de tipo seletiva. Por isso, cabe uma reflexão sobre a relevância dessas habilidades em geral na vida acadêmica. A atenção tem papel importante para a percepção, linguagem e memória, sendo o fator de maior influência na aprendizagem^(24)^. A atenção sustentada mantém a atenção do estudante durante toda a atividade realizada, como ao ler um texto, mesmo que haja fatores distratores^(23)^. Já a memória possibilita a análise e a aquisição de novos conhecimentos e posterior aplicação em outros contextos, assim como permite o desenvolvimento de diversas atividades^(24)^.

A dificuldade em memorizar tarefas que foram apenas ouvidas foi, também, uma das queixas de maior prevalência neste estudo, e está relacionada à memória auditiva^(25)^. Alterações na memória auditiva podem levar à utilização imprópria da linguagem oral e escrita, dificuldade na compreensão do discurso de outra pessoa e, consequentemente, transtornos no aproveitamento escolar e na integração com os pares^(25)^. Essa habilidade desempenha grande relevância na retenção e recordação dos aprendizados e, por isso, é importante que seja estimulada em ambientes escolares e no cotidiano dos estudantes^(25)^.

Por fim, pode-se perceber que as queixas de domínio auditivo mais prevalentes neste estudo apresentaram associação com queixas relacionadas às funções cognitivas de memória, atenção e planejamento, tal como a ausência de queixas auditivas esteve associada à ausência das queixas envolvendo habilidades cognitivas. Isso pode ser justificado ao se notar que algumas habilidades do Processamento Auditivo Central dependem de outras funções cognitivas, como atenção, memória, linguagem, funções executivas, entre outras^(26,27)^.

Um estudo^(27)^ observou que as funções executivas compartilham mecanismos cognitivos subjacentes às habilidades auditivas, especialmente em testes que investigam atenção concentrada, percepção de faces, linguagem oral e memória (de trabalho e episódico-semântica). Para mais, há evidências de que, apesar de existirem algumas regiões do cérebro específicas para estímulos de modalidade auditiva, o processamento de dados sensoriais é interdependente e integrado com base em domínios cognitivos, especialmente atenção, memória e representações linguísticas^(27)^, o que justifica as associações encontradas.

É importante destacar que o presente artigo apresenta resultados aplicáveis à população estudada e apresenta limitações como o fato de o questionário ser autorreferido. Dessa forma, sugere-se que novas pesquisas sejam realizadas associando os resultados obtidos no Questionário a bateria de testes do Processamento Auditivo Central e a protocolos validados para avaliação de habilidades de domínio acadêmico.

Ademais, um estudo^(28)^ demonstrou que há associação entre aspectos comportamentais e a motivação para aprender. Sendo assim, é necessário que esses fatores sejam considerados em futuras pesquisas, uma vez que podem influenciar nas respostas encontradas.

Devido ao fato deste estudo buscar descrever as queixas auditivas e acadêmicas de adultos estudantes e servidores de uma universidade pública em geral, optou-se por não adotar como critérios de exclusão: perdas auditivas de qualquer tipo e grau e alterações sindrômicas, neurológicas e/ou cognitivas. Todavia, é interessante que, num futuro estudo, esses critérios sejam considerados, visto que essas condições podem interferir nas queixas apresentadas e resultados obtidos. Faz-se relevante, ainda, controlar o fator idade em pesquisas posteriores, investigando os indivíduos por faixa etária.

Como contribuições, o estudo evidenciou associações estatisticamente significantes entre funções cognitivas e habilidades auditivas autorreferidas que se somam a pesquisas anteriores e sustentam estudos futuros. A pesquisa favorece ainda a elaboração de planejamentos educacionais e de intervenção, ao demonstrar a relevância das funções executivas na vida acadêmica, especialmente no Ensino Superior, que devem ser consideradas para um melhor aproveitamento estudantil e melhor prognóstico terapêutico.

À vista disso, é relevante que esses fatores sejam considerados ao realizar avaliações do Processamento Auditivo Central; ao se intervir em pacientes com queixas auditivas, certificando-se da presença ou ausência de fatores cognitivos associados; e na vida estudantil, buscando proporcionar aos alunos um ambiente auditivo adequado para o aprendizado e identificando possíveis lacunas no desenvolvimento da cognição que devem ser exercitadas para possibilitar a aquisição plena de conhecimento.

## CONCLUSÃO

Os resultados encontrados evidenciam dificuldades auditivas e acadêmicas de adultos universitários e graduados, contendo como queixas mais prevalentes a dificuldade acadêmica relacionada à concentração, à memória e ao planejamento, e as dificuldades auditivas de compreender no ruído e memorizar tarefas que foram apenas ouvidas.

Foi possível observar, ainda, que há associação entre queixas auditivas e acadêmicas em adultos, marcada pela relação de aspectos cognitivos com aspectos auditivos.
